# Structures of HIV-1 Neutralizing Antibody 10E8 Delineate the Mechanistic Basis of Its Multi-Peak Behavior on Size-Exclusion Chromatography

**DOI:** 10.3390/antib10020023

**Published:** 2021-06-10

**Authors:** Young Do Kwon, Xiangchun E. Wang, Michael F. Bender, Rong Yang, Yile Li, Krisha McKee, Reda Rawi, Sijy O’Dell, Nicole A. Schneck, Andrew Shaddeau, Baoshan Zhang, Frank J. Arnold, Mark Connors, Nicole A. Doria-Rose, Peter D. Kwong, Q. Paula Lei

**Affiliations:** 1 Vaccine Research Center, National Institute of Allergy and Infectious Diseases, National Institutes of Health, Bethesda, MD 20892, USA; ydkwon@mail.nih.gov (Y.D.K.); michael.bender2@nih.gov (M.F.B.); mckeek@mail.nih.gov (K.M.); reda.rawi@nih.gov (R.R.); odells@mail.nih.gov (S.O.); baoshan.zhang@nih.gov (B.Z.); nicole.doriarose@nih.gov (N.A.D.-R.); 2 Vaccine Production Program, Vaccine Research Center, National Institute of Allergy and Infectious Diseases, National Institutes of Health, Gaithersburg, MD 20878, USA; xiangchun.wang@nih.gov (X.E.W.); rong.yang@nih.gov (R.Y.); yile.li@nih.gov (Y.L.); nikki.schneck@nih.gov (N.A.S.); andrew.shaddeau@nih.gov (A.S.); Drfrank.arnold@yahoo.com (F.J.A.); 3 HIV-Specific Immunity Section of the Laboratory of Immunoregulation, National Institute of Allergy and Infectious Diseases, National Institutes of Health, Bethesda, MD 20892, USA; mconnors@niaid.nih.gov

**Keywords:** cis-trans proline isomerization, conformational isomerization, hydrophobic interaction chromatography (HIC), mobile phase modifier, monoclonal antibody 10E8, MPER, secondary matrix interaction, size-exclusion chromatography (SEC)

## Abstract

Antibody 10E8 is capable of effectively neutralizing HIV through its recognition of the membrane-proximal external region (MPER), and a suitably optimized version of 10E8 might have utility in HIV therapy and prophylaxis. However, 10E8 displays a three-peak profile on size-exclusion chromatography (SEC), complicating its manufacture. Here we show cis-trans conformational isomerization of the Tyr-Pro-Pro (YPP) motif in the heavy chain 3rd complementarity-determining region (CDR H3) of antibody 10E8 to be the mechanistic basis of its multipeak behavior. We observed 10E8 to undergo slow conformational isomerization and delineate a mechanistic explanation for effective comodifiers that were able to resolve its SEC heterogeneity and to allow an evaluation of the critical quality attribute of aggregation. We determined crystal structures of single and double alanine mutants of a key di-proline motif and of a light chain variant, revealing alternative conformations of the CDR H3. We also replicated both multi-peak and delayed SEC behavior with MPER-antibodies 4E10 and VRC42, by introducing a Tyr-Pro (YP) motif into their CDR H3s. Our results show how a conformationally dynamic CDR H3 can provide the requisite structural plasticity needed for a highly hydrophobic paratope to recognize its membrane-proximal epitope.

## 1. Introduction

Since the first officially reported case of AIDS in 1981, the HIV-1 pandemic has become one of the severest public health crises in modern times [1,2,3]. After the introduction of combinations of antiretroviral medications in the 1990s, HIV-1 infection has been transformed from a fatal disease into a chronically manageable one [4,5,6,7,8]. More recently, broadly neutralizing monoclonal antibodies (bNAbs) have been identified with highly specific recognition of the HIV-l envelope (Env) glycoprotein, and these are providing additional opportunities to treat or to prevent HIV-1 infection.

Antibody 10E8 is one of the broadest monoclonal antibodies thus far identified that effectively neutralizes diverse strains of HIV-1 [9,10,11]. It targets the membrane-proximal external region (MPER) of the HIV-1 viral spike with a highly hydrophobic antigen-binding region [12]. This hydrophobicity, which is critical to the potency of 10E8, also imparts production challenges with respect to poor solubility in aqueous solution and anomalous chromatographic behavior [11].

Size-exclusion chromatography (SEC) provides separation based on hydrodynamic size and shape of the molecule to measure protein aggregation during clinical product development [13,14,15]. During clinical development of 10E8, traditional SEC was used to evaluate aggregation, a critical quality attribute. 10E8 and two variants, 10E8v4, a solubility enhanced variant with 26 amino acids changes, [11] and 10E8VLS [10], a variant with solubility and potency enhanced version of 10E8 (Summarized in Table S1), manifested multi-peak SEC profiles. Since this atypical SEC behavior prevents aggregation assessment, both a revised SEC method and further understanding of the root cause were needed. In a separate study, we report a newly developed fit-for-purpose SEC method that overcomes this multi-peak challenge [16]. By using a combination of modifiers (100 mM arginine at high pH 10.55), the new SEC method could successfully merge all monomeric populations into one, thereby allowing separation of a well-quantified aggregate peak. Notably, the modifiers (high pH and arginine) could not achieve the desired merger independently.

An understanding of the mechanistic basis of the multi-peak profile and the modifier-induced merging of peaks from the traditional SEC method is critical to gaining insight into the molecular structure of 10E8 and its correlated function. Masiero et al. and Guttman et al. recently reported that the SEC anomaly of 10E8 is attributed to the Tyr-Pro-Pro (YPP) motif in the CDR H3 [17,18]. However, these two papers do not agree upon which specific residue is responsible for the anomaly and a structural definition of different conformational states of the CDR H3 has also been missing. Here we report the use of a combination of structural methods, mutational analyses, and biophysical assessments to investigate the cause of the multipeak profile and its resolution through use of synergistic modifiers. We employed an optimized hydrophobic interaction chromatography (HIC) method to understand the different levels of hydrophobicity and their impact on the SEC profile [19,20]. We also defined multiple hydrophobic states of an antibody, their root cause, and their effects on SEC, and show that this provides a basis for the fit-for-purpose SEC method. Further, we determined crystal structures of alanine mutants of a critical YPP motif, to understand the conformational basis of both multiple peaks and their influence on recognition, investigated the impact of the YP motif on other MPER-directed antibodies, and compared and contrasted our results with the recently published studies [17,18]. Overall, these results provide atomic-level details for how a functionally critical cis-proline can alter the chromatographic characteristics of an antibody and can assist in the recognition of a hydrophobic target.

## 2. Materials and Methods

### 2.1. Chemicals and Reagents

The monoclonal antibodies (mAbs) 10E8 [9] and its variants, 10E8v4 [11], 10E8v4-100cF [10] and 10E8v4-5R+100cF (also known as 10E8VLS) [10], were provided by the research development laboratories of the Vaccine Research Center (VRC/NIAID/NIH, Bethesda and Gaithersburg, MD, USA). l-arginine and ammonium sulfate were purchased from J.T. Baker (Center Valley, PA, USA), papain from Pierce^TM^ (Thermo Fisher Scientific, Waltham, MA, USA), Lys-C from New England Biolabs (Beverly, MA, USA), Na phosphate monobasic from EMD (Gibbstown, NJ, USA) and ten-fold (10×) PBS from Lonza (Walkersville, MD, USA). LC/MS grade water (Avantor Performance Materials, Center Valley, PA, USA) was used in all sample preparation procedures.

### 2.2. Production of 10E8 CDR H3 Variants

Proline mutations of 10E8v4 at residues 100f and 100g (Kabat numbering) in the CDR H3, P100fA and P100gA were introduced into the 10E8v4 heavy chain plasmid. 10E8v4 with a disulfide bond locked CDR H3 was generated by introducing Cys mutations at positions 100e in the 10E8v4 heavy chain and 30 in the 10E8v4 light chain. T CDR H3-deleted (_99_YDFWSGYPP_100g_) version of 10E8v4 was produced by overlapping PCR mutagenesis using to 10E8v4 heavy chain expression plasmid.

To express the variants, Expi293F cells (Thermo Fisher) were cotransfected with the expression vectors encoding the heavy and the light chain of the variants using Turbo293 transfection reagent (SPEED BioSystems, Gaithersburg, MD, USA). After 6 days post-transfection, antibody expressed supernatants were harvested and purified with a Protein A (GE Healthcare, Chicago, IL, USA) column. Antibodies were eluted with Pierce IgG elution buffer (Thermo Fisher Scientific, Waltham, MA, USA), neutralized with 1 M Tris-HCl, pH 8, and dialyzed against PBS, pH 7.4.

### 2.3. Generation of Antigen-Binding Fragment (Fab) of 10E8v4 and 10E8VLS

10E8v4 Fab was generated by endoproteinase Lys-C digestion as described previously [9]. Briefly, the digestion was performed in a reaction mixture containing Lys-C and antibody (1:2000 (*w*/*w*)) in 50 mM Tris-HCl, pH 8.0 at 37 °C overnight. The mixture was then dialyzed against PBS, pH 7.4 for 2–3 h at 22 °C and run over a Protein A column to remove Fc fragments and undigested IgG. 10E8VLS Fab was generated by papain digestion of 10E8VLS (1:1000 (*w*/*w*)) in a reaction mixture containing 100 mM sodium phosphate, 50 mM NaCl, 30 mM cysteine and 2 mM EDTA, pH 6.4 for 45 min at 37 °C. The digestion mixture was subsequently passed through a Protein A cartridge to remove Fc and undigested IgG, and the buffer was exchanged with 2× PBS using a 30 kDa cutoff Amicon filter. The collected Fab peak 1 and peak2 were tested by SEC at Time 0, Time 24 h RT and Time 24 h 4 °C.

### 2.4. Biolayer Interferometry

The kinetics of 10E8v4 variants and gp41 peptide bindings were measured by Octet (ForteBio). Ni-NTA biosensors (Molecular Devices), presoaked in PBS for 5 min, were loaded with T117-F MPER scaffold [21] to the density of 0.4 nm. Then, the association of 10E8v4, 10E8v4-100gA, 10E8v4-100gA or 10E8v4-100fgAA was measured by dipping the MPER scaffold-loaded biosensors into microplate wells containing Fabs of 10E8v4 variants in series of concentrations, ranging from 15.6 to 1000 nM in 2-fold dilutions in HBS-EP+ buffer (GE Healthcare) for 5 min, and the dissociation was measured by dipping the biosensors into wells containing HBS-EP+ buffer for 1 min. The kinetic parameters were extracted by fitting globally the entire association and the dissociation for 5 sec with a 1:1 Langmuir binding model.

### 2.5. Expression, Purification, Crystallization and Structure Determination

The plasmids encoding 10E8v4 variant heavy chain genes and plasmids encoding corresponding light chain genes were mixed with Turbo293 transfection reagent (SPEED BioSystems), added to 100 mL of 293 EXPI cells (Thermo Fisher) at 2.5 × 10^6^ mL and incubated in a shaker incubator at 120 rpm, 37 °C, 9% CO_2_. At 5 days post-transfection, cell culture supernatant was harvested, and the expressed 10E8v4 variants were purified with a Protein A column. The purified IgGs were then digested with Lys-C (New England BioLabs) to make Fabs, according to the manufacturer’s protocol, followed by another round of a protein A column purification and Superdex 200 HiLoad 16/600 (GE Healthcare) size exclusion chromatography. The Fabs in 5 mM HEPES, 7.4, 150 mM NaCl, were concentrated to 15 mg/mL and were screened for crystallizing conditions with in-house Hampton Research, Precipitation Synergy, and Wizard screen using a Mosquito robot (TTP Labtech). Initial hits were optimized with hanging-drop vapor-diffusion method. The mother liquor comprised of 12% PEG 8K and 0.1 M Tis-HCl, pH 8.5 or 3.6 M Na formate and 0.1 M Na acetate, pH 4.6 yielded diffracting quality crystals of 10E8v4-100fA. The 20% PEG 8K 0.1 M Tris-HCl, pH 8.5 yielded crystals of 10E8v4-100gA, 10% PEG 8K 0.2 M, 5% iso-propanol and ammonium sulfate 0.1 M HEPES, pH 7.4 yielded crystals of 10E8v4-100fgAA. Diffracting data were collected from a single crystal soaked and frozen in cryoprotectants containing 30% glycerol with mother liquor solutions at a synchrotron beamline (SER-CAT ID22, APS) and scaled with HKL2000 [22]. The structures were solved by molecular replacement using Phaser [23] and 10E8v4 structure in complex with gp14 (PDB ID:5IQ9) as a search model, built with COOT [24] and refined with PHENIX [25].

### 2.6. Neutralization Assays

Single-round-of-replication Env pseudoviruses were prepared, titers were determined and the pseudoviruses were used to infect TZM-bl target cells as described previously [26]. Neutralization of monoclonal antibodies was determined using a multiclade panel of 9 HIV-1 Env-pseudoviruses including clade A (2), clade AE (1), clade B (3) and clade C (3). Each mAb was assayed at 5-fold dilutions starting at 50 µg/mL. The neutralization titers were calculated as a reduction in luminescence units compared with control wells and reported as 50% or 80% inhibitory concentration (IC50 or IC80) in micrograms per milliliter.

### 2.7. Traditional Size Exclusion Chromatography

Traditional SEC method was executed on the Waters Acquity UPLC system and Acquity UPLC Protein BEH SEC column (200 Ă, 1.7 µm, 4.6 mm × 150 mm, Waters, Milford, MA, USA) at an isocratic flow rate of 0.4 mL/min with 2× PBS mobile phase. Running time of each sample was 12 min and was extended for proteins that undergo non-specific interactions with the column. The detection was UV absorbance at 280 nm.

### 2.8. Product-Specific Size Exclusion Chromatography

In summary for the product-specific method, running buffer was optimized at 2× PBS and 100 mM arginine with a final pH of 10.55 and a running time of 6 min. Additional details are described in [16].

### 2.9. Optimized Hydrophobic Interaction Chromatography

HIC was performed on a Waters Acquity UPLC system with ProPac^TM^ HIC 10 LC column of 300 Ă, 5 µm and 4.6 mm × 250 mm (Thermo Fisher, Sunnyvale, CA, USA) at a flow rate of 1 mL/min. 10E8 variants did not elute from the column under the default HIC method described in the supplier’s insert. An optimized HIC condition used to elute 10E8 variants. The optimized conditions utilized Eluent A at 125 mM (NH_4_)_2_SO_4_, 25 mM NaH_2_PO_4_, with 100 mM arginine at a final pH of 9.1 and Eluent B at 25 mM NaH_2_PO_4_ with 100 mM arginine at a final pH of 10.1; the gradient was (1): 0–2 min, 100% A; (2) 2–12 min 100% A to 40% A 60% B; (3) 12–62 min 40% A 60% B to 100% B. Conversion studies of individual fractions are included in the supplement information (SI).

## 3. Results

### 3.1. CDR H3 of 10E8 Variants Is the Cause of Both SEC Heterogeneity and Extended Column Interactions

**Figure 1:**
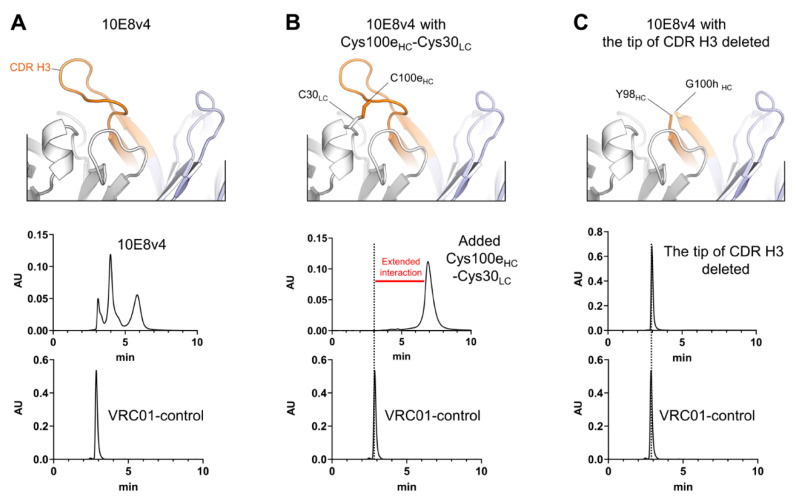
CDR H3 of 10E8 is the cause of extended interaction with SEC. (**A**) Top panel, crystal structure of 10E8v4 (PDB ID:5IQ9); bottom panels, anomalous SEC profile of 10E8v4 versus antibody VRC01 control. (**B**) After introducing a disulfide bond by mutation of CDR H3 (Tyr100eCys) and light chain (Ser30Cys), a single peak of the 10E8v4 modified Cys100e_HC_-Cys30_LC_ was observed, but with anomalously longer SEC profile compared to antibody VRC01. Top panel, model of the disulfide bond linked CDR H3; bottom panels, SEC chromatography of 10E8 variant highlighting anomalous SEC interaction versus antibody VRC01 control. (**C**) Deletion of the tip of CDR H3 (_99_YDFWFGYPP_100g_) leads to loss of extended SEC interactions. Top panel, model of the 10E8v4 structure with the tip of CDR H3 deleted; bottom panels, SEC chromatography of 10E8 variant highlighting anomalous SEC interaction versus antibody VRC01 control.

We previously showed formation of a disulfide bond between light chain residue Cys30 and heavy chain Cys100e of 10E8v4 to result in a single SEC peak [11]. Here we show the disulfide bond peak to run anomalously longer on SEC, when compared to antibody VRC01 (control), a well characterized HIV-1 neutralizing antibody that behaves as an expected monoclonal antibody by SEC [27] (Figure 1A,B). Further, we found that deletion of the tip of the CDR H3 (YDFWSGYPP) also resulted in a variant of 10E8v4 that eluted as a single SEC peak. In the case of the CDR H3 deletion, the SEC peak eluted at the expected position of a monomer and was not retained anomalously longer on the SEC column (Figure 1C). These results indicated the anomalous peak profiles and retained positions of peaks on SEC to be a result of interactions of the hydrophobic CDR H3 of 10E8v4 with the separation matrix, while stabilization of the active conformation of the CDR H3, as fixed by the Cys30-Cys100e disulfide bond, resulted in a single peak, with extended interactions with the column.

### 3.2. Two Predominant Slowly Interconverting Conformations of Fab Explain Three Monomeric Population Profile of 10E8 Immunoglobulin (IgG)

**Figure 2:**
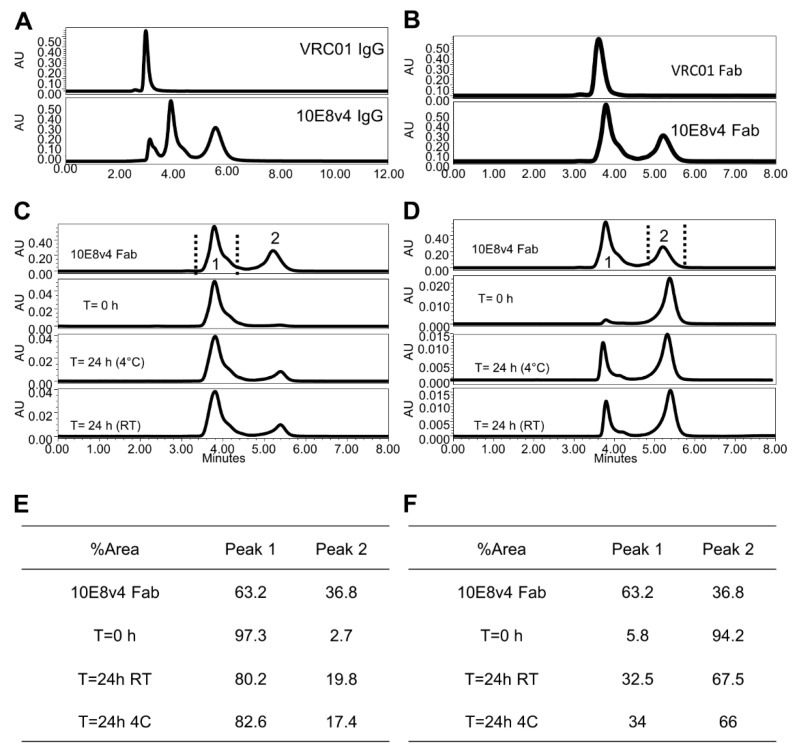
10E8v4 IgG displays a three-peak profile, whereas Fab displays a two-peak profile. (**A**) Three-peak profile of 10E8v4 IgG with VRC01 as the control. (**B**) Two-peak monomeric population profile of 10E8v4 Fab. (**C**) SEC profile of times-dependent reassortment of 10E8v4 Fab fractionation of Peak 1. (**D**) SEC profile of time-dependent reassortment of 10E8v4 Fab fractionation of Peak 2. (**E**) Tableted redistribution of 10E8v4 Fab Peak 1 fractionation upon injection time and temperature conditions. (**F**) Tableted redistribution of 10E8v4 Fab Peak 2 fractionation upon injection time and temperature conditions.

The disulfide bond-fixed variant of 10E8 was not as potent on some isolates as the potency-improved 10E8VLS, so while identifying the source of the heterogeneity, this variant did not allow us to side-step the three-monomeric population profile challenge of 10E8VLS. To provide a mechanistic understanding of this three-population behavior, we first analyzed the more soluble 10E8v4 [11], as this variant had better solubility and showed the same three-monomeric population profile as the 10E8VLS variant in preclinical development (Figure 2A), with the first peak eluting at the expected monomeric position, and with two additional peaks eluting anomalously longer; separation of these peaks resulted in dynamic redistribution of the three peaks [11].

As the root cause to the three-population anomaly was related to the CDR H3, we sought to characterize the SEC behavior of the antigen-binding fragment (Fab) of 10E8v4, as the monovalent Fab only has a single CDR H3, not the two CDR H3s of the bivalent immunoglobulin. SEC of the 10E8v4 Fab showed a predominant two-peak monomeric population profile, with the first peak (Peak 1) eluting at a monomeric position (as VRC01), and an additional peak (Peak 2) eluting anomalous longer (Figure 2B). While the positions of the three 10E8v4 peaks were consistent with the single and double retention of the two Fabs peaks, the prevalence of the peaks was slightly different: the observed ratio of Fab Peak 1 and Peak 2 was approximately 60:40, whereas deconvolution of the three 10E8v4 peaks of the immunoglobulin was consistent with an approximate 40:60 ratio. Separation of Fab peaks showed the same dynamic redistribution as the parent 10E8v4 [11] (Figure 2C,D), with the distribution of Fab Peak 1 and Peak 2 exhibiting a trend to return to the starting profile (Figure 2E,F).

We also analyzed a variant of 10E8v4 with two mutations, Arg at residue 5 and Phe at residue 100c, both on the heavy chain. This variant, named 10E8VLS, showed a 10-fold increase in potency [10]. The 10E8VLS antibody, which has progressed to product development for clinical application and is further discussed as part of this study. Like 10E8v4, the 10E8VLS variant showed a predominant three-peak population on SEC (Figure S1A), though the first two peaks were of lower prevalence than that of 10E8v4 and with longer retention time than VRC01 and 10E8v4 (see Figure 2A, SEC for 10E8v4, with the first eluted peak showing a similar retention time as VRC01). All peaks of 10E8VLS were confirmed to be of monomer mass by SEC-MALS. The 10E8VLS Fab (Figure S1B) showed predominately two peaks on traditional SEC, with one most abundant portion more than 80% (Peak 2) eluted at the latest retention time. The main Fab Peak 2 was collected and reassessed for re-equilibrium by traditional SEC after 24 h at 4 °C and room temperature (RT) (Figure S1D). In both temperature conditions the distribution of Fab Peak 2 returned to the starting profile (Figure S1C summarized 10E8VLS Fab percent distributions), demonstrating the existence of an equilibrium of different hydrophobicity for the 10E8VLS Fabs, consistent with the phenomenon observed for 10E8v4.

Overall, two predominant SEC peaks were observed for Fab 10E8v4 and 10E8VLS. These likely corresponding to two predominant conformations of the CDR H3. Three SEC peaks, moreover, appeared to combine in the IgG context to yield 3 peaks, thereby providing an explanation for the observed multi-peak monomeric population profile of 10E8 and its variants (see schematic shown in Figure S2).

### 3.3. 10E8VLS Immunoglobulin Exists in at Least Three Equilibrium States, Each with Different Hydrophobicity

Based on insight gained into the SEC profile of 10E8, we hypothesized the CDR H3 of 10E8 would present an equilibrium of conformational structures, each with a different degree of hydrophobic surface exposure leading to different degrees of hydrophobic interaction with various column matrices [11]. To test this hypothesis, the hydrophobicity of the 10E8 product, 10E8VLS, was examined by a HIC method in which molecules are differentiated by the strength of their hydrophobic interactions with the column resin.

By utilizing an optimized HIC method, VRC01 eluted at the void time, again demonstrating much lower hydrophobicity (Figure S3A). 10E8VLS eluted later in three major populations (Figure S3B), with a similar profile as that in the traditional SEC profile of 10E8VLS (Figure S3C). Thus, three hydrophobic states were clearly present and confirmed for 10E8VLS. The similar profile distributions indicated the three-peak population distribution observed in the traditional SEC to be the result of the secondary interactions of hydrophobicity between the 10E8VLS and the SEC column matrix. The correlation of this similar three-elution profile was further studied by fractionation of each peak.

To aid in further understanding the correlation of SEC profile and hydrophobicity, the same 10E8VLS material was subjected to SEC and HIC analysis in parallel. 10E8VLS was separated and fractionated by the traditional SEC method and individual peaks were then reinjected side-by-side to both SEC and HIC (Figure S4 A1, A2 and A3 by SEC and B1, B2 and B3 by HIC correspondingly). The order and profile observed by HIC for individual fraction correlated strongly with the order and profile run on the traditional SEC method. Overall, the HIC correlation study further supports our findings that 10E8VLS exhibits at least three different hydrophobic states, resulting in various levels of secondary interaction between 10E8VLS and the SEC column matrix, and the strength of secondary interaction depends upon the hydrophobic state.

**Figure 3:**
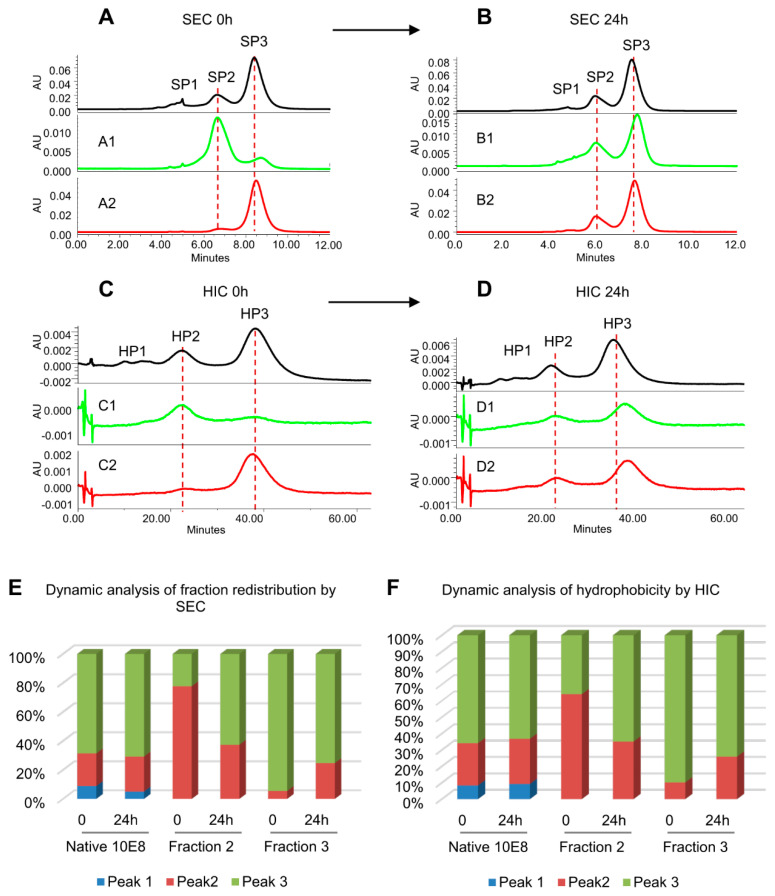
Conversion of isolated and reinjected 10E8VLS fractions from SEC (A’s and B’s) and HIC (C’s and D’s) at time 0 and after 24 h. (**A**) SEC profiles of 10E8VLS neat injection at T0. A1 and A2: collected fraction 2 and 3 by SEC and reinjected to SEC column at T0. (**B**) SEC profiles of 10E8 native after 24 h incubation at 25 °C. B1 and B2: collected fraction 2 and 3 by SEC and reinjected to SEC analysis after 24 h incubation at 25 °C. (**C**) HIC profiles of 10E8 native at T0. C1 and C2: collected fraction 2 and 3 by SEC and reinjected to HIC column at T0. (**D**) HIC profiles of 10E8 native after 24 h incubation at 25 °C. D1 and D2: collected fraction 2 and 3 by SEC and reinjected to HIC analysis after 24 h incubation at 25 °C. (**E**) Bar graph of conversion trend after fractions being reinjected to SEC column. (**F**) Bar graph of the conversion trend after fractions being reinjected to HIC column.

Further studies were carried out to evaluate the conformational interconversion of 10E8VLS peaks by both SEC and HIC analysis (Figure 3) SEC fractions (SP2 and SP3) were collected and incubated at room temperature for 24 h. The SEC and HIC profile for each fraction at Time 0 and 24 h were collected. For each SEC fractions, the change of HIC profile over 24 h incubation followed the same trend as the change of SEC profile. In Figure 3, the SEC (Figure 3A,B) and HIC (Figure 3C,D) of 10E8VLS fractions incubated at room temperature for 24 h are displayed in the order of fraction 1, fraction 2 and fraction 3. For fraction SP2, comparing to time 0, the SEC profile of fraction SP2 had a clear shift towards SP3. A similar trend of shifting is observed for the HIC elution profile of fraction 2. In addition, a shift towards SP2 and HP2 are observed for SEC and HIC data obtained for fraction 3, respectively (Figure 3B,D). The trend for each fraction is plotted in the bar graph (Figure 3E,F). The integrated area under each peak (Peak 1, 2 and 3) at time 0 and 24 h for fraction 1, 2 and 3 are represented by blue, orange and gray, respectively. Numerical data confirmed the synchronized conversion of SEC and HIC profile (Figure 3E,F). Thus, at time 0 and 24 h, the HIC and SEC results were well correlated. Overall, the results indicated that there exists a dynamic equilibrium of three-hydrophobic states, with HIC data providing evidence for hydrophobic interactions providing a basis for the observed multi-peak profiles.

### 3.4. Use of Modifier Arginine with Optimized pH Coeffectively Masks Secondary Interactions between 10E8 and Column Matrices

Typical SEC separates biomolecules by apparent hydrodynamic size (size and shape in solution) and delivers a measurement of aggregation after separation. However, the secondary interactions of 10E8VLS with the SEC packing sorbents can be sufficiently strong as to make the goal of separating and quantifying aggregates challenging. Therefore, it can be necessary to find an effective modifier to mitigate this interaction [28,29].

Arginine is known to improve separations of aggregates in proteins and can minimize secondary interactions by reducing the hydrophobic surface exposure [30]. One mechanism relates to the stacking of arginine in a head-to-tail manner: arginine in solution displays a hydrophobic surface by aligning its three methylene groups, and this hydrophobic surface of arginine can interact with the hydrophobic residues on the protein surface to mask protein surface hydrophobicity [31]. We therefore speculated that the effect of arginine in our product specific SEC method could be attributed to this masking effect. The three major monomeric population elution times moved closer together with higher concentrations of arginine, supporting the hypothesis of arginine as a workable modifier [16].

We found 100 mM arginine and pH 10.55 must act collaboratively to completely merge the three-population profile of 10E8 by a product-specific SEC method [16]. A limited effect of single modifiers (either arginine or pH) on the HIC profile was also observed (Figure S5). This data showed that in the neutral and non-arginine solution, 10E8VLS has multimeric hydrophobic states, as demonstrated through the HIC profile; only at pH 10.55 with 100 mM arginine condition, did the different hydrophobicities minimize and 10E8VLS elute as one peak. The gradual merging of 10E8VLS multiple peaks in HIC with increasing pH from 10 to 10.55 in the presence of 100 mM arginine (Figure S6) was consistent with peak merging observed in SEC under similar arginine and pH conditions [16].

A possible mechanism for the specific pH requirement for the product-specific SEC may relate to the large number of tyrosine (side chain pKa 10.1) in the 10E8 CDR H3. At pH 10.55 with arginine, the 10E8 CDR H3 would be substantially negatively charged, while arginine would still be positively charged (pI of 10.78) [32,33,34]. Attraction between the negatively charged 10E8 CDR H3 and the positively charged arginine may reach a sweet spot at the observed optimized pH of 10.55 (±0.1), facilitating the formation of a uniformed apparent hydrodynamic radius with stacked arginine clusters shielding hydrophobic 10E8 CDR H3 surfaces.

To further support this hypothesis, the effects of other amino acids, such as lysine, histidine and proline, were also evaluated as potential modifiers in SEC mobile phase (Figure S7). Among these amino acids, lysine was found to have a similar merging effect on 10E8VLS under the same pH condition. Lysine, with a side chain’s pKa higher than 10.55, would be expected to have a similar charge effect as arginine; interestingly and similarly, lysine also has four methylene groups on its side chain, which could align with 10E8VLS hydrophobic patches in the similar way as arginine, thereby resulting in a shielding effect that minimized secondary hydrophobic interactions with the SEC column. Other tested amino acids did not have both characteristics and therefore would not be expected to have this synergic effect as modifiers for the 10E8VLS product-specific SEC method. Overall, for arginine (or lysine), pH functioned as an adjustable value, regulating the arginine masking of 10E8 CDR H3 hydrophobic surface patches.

### 3.5. Modification of the Di-Proline Motif in the 10E8 CDR H3 Alters Both SEC and HIC Profiles

The CDR H3 of 10E8 contains a proline–proline motif (residues 100f–100g, Kabat numbering) [35], which are connected by a cis-peptide bond (PDB ID 4G6F) [9,11]. Slow cis-trans conformational isomerization has been shown to play a key role as a rate determining step of protein folding [36] and has been implicated in minute-time scale governance of antibody recognition of a viral antigen [37]. Di-proline motifs, moreover, have been found to exhibit in some cases even slower conformational isomerization [38], and thus might provide the underlying molecular basis for the CDR H3 conformational heterogeneity we observed with antibody 10E8.

**Figure 4:**
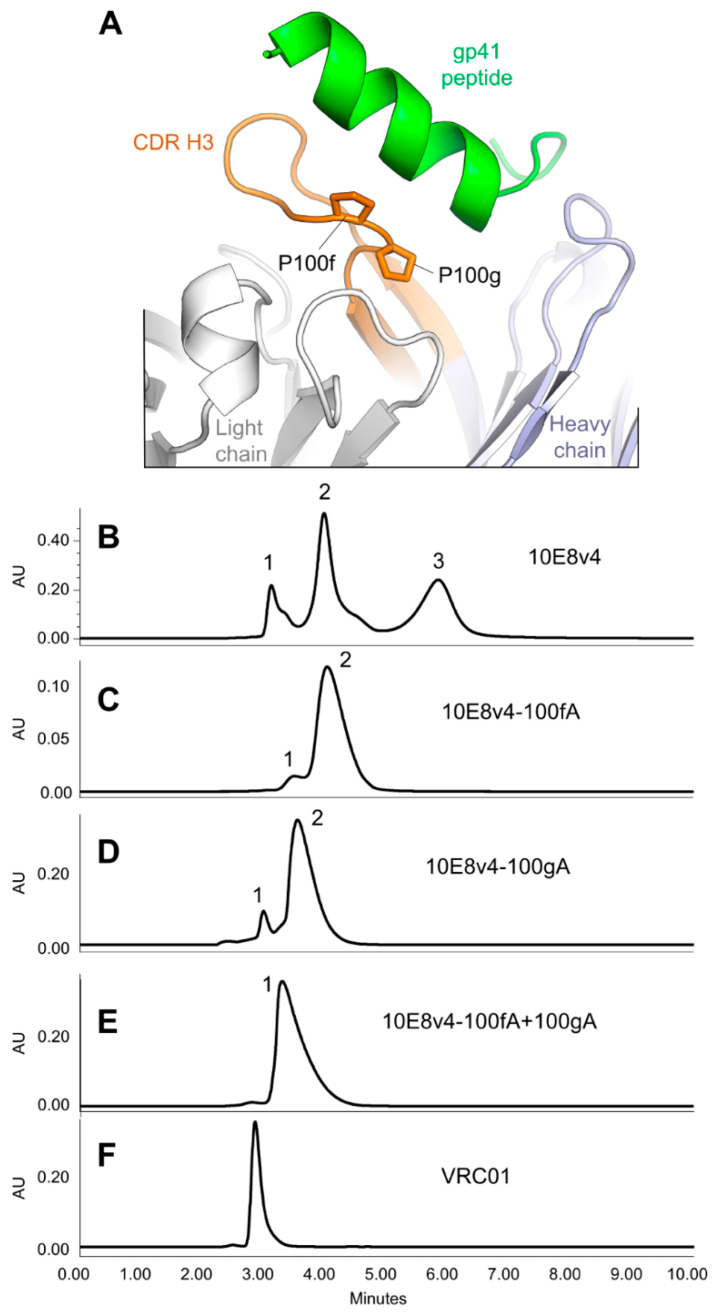
Modifications of a di-proline motif in the CDR H3 alter the SEC profile. (**A**) Prolines at residues 100f and 100g are highlighted in stick representation (Kabat residue numbering is used for antibody). (**B**) The SEC profile of 10E8v4. (**C**) The SEC profile of 10E8v4-P100fa. (**D**) The SEC profile of 10E8v4-P100gA. (**E**) The SEC profile of 10E8v4-P100fA+P100gA. (**F**) The SEC profile of VRC01.

We tested the influence of the di-proline motif in the CDR H3 of 10E8v4 on its SEC and HIC profiles by mutating prolines at positions 100f or 100g to alanine (Figure 4A). Compared to the SEC of 10E8v4 (Figure 4B), mutating either of these prolines resulted in the three-peak profile of 10E8 merging into a predominantly single peak (Figure 4C–E), suggesting that the prolines cause the SEC anomaly.

### 3.6. Crystal Structures of the YPP Motif Variants of 10E8 Reveal the Degree of Structural Compatibility to Trend with Binding Affinity and Neutralization Potency

**Figure 5:**
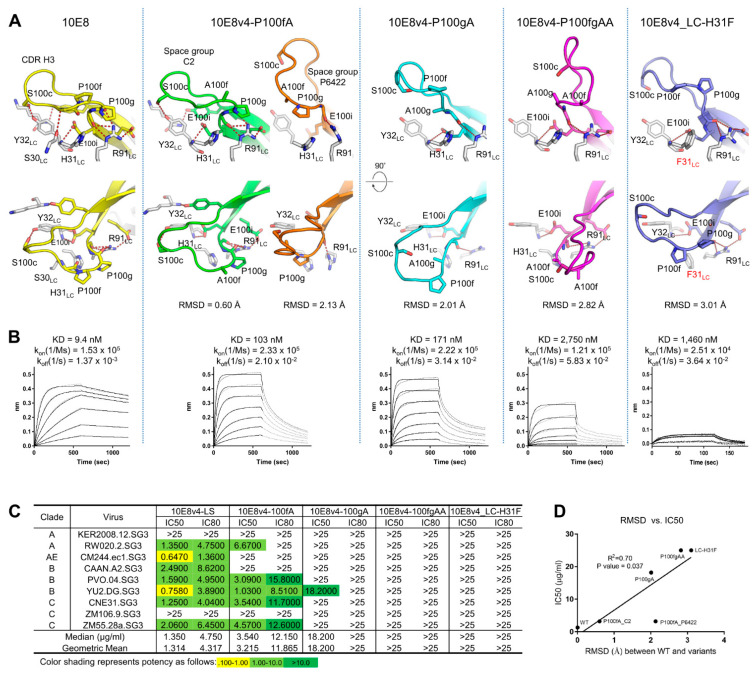
Active conformation similarity of 10E8 variants involving CDR H3 di-proline motif mutants or motif-interacting residues correlates with neutralization potency. (**A**) Different conformations of the CDR H3s were shown with RMSDs of Cα between the variants and 10E8 structure. Hydrogen bonds between the residues in the CDR H3 and the light chains were shown in dotted lines. (**B**) Binding kinetics of Fab molecules of 10E8v4 variants to T117-F MPER scaffold measured by BLI. (**C**) Neutralization potency and breadth of 10E8v4 variants against select viruses. (**D**) Correlation between RMSD and median IC50.

To define the structural basis for the anomalous SEC and HIC behavior, we determined the crystal structures of 10E8v4 with Pro100f to Ala (10E8v4-100fA) in two different space groups, Pro100g to Ala (10E8v4-100gA) and Pro100f and Pro100g to Ala (10E8v4-100fgAA) variant at 3.7 Å, 2.4 Å, 2 Å and 2.7 Å resolutions, respectively (Figure 5 and Table 1). 

The structures were solved by molecular replacement using PDB ID:5IQ9 as a search model. After rigid body refinement, the CDR H3s (_95_TGKYYDFWFGYPPGEE YFQD_102_) of the variants were defined clearly in their *Fo-Fc* maps contoured at 3σ (Figure S8A). When the CDR H3s of the refined structures were superimposed, they were noticeably different from that of the ligand-free 10E8 (PDB ID:5JNY) with RMSDs of 0.60 Å, 2.13 Å, 2.01 Å and 2.82 Å for 10E8v4-P100fA (space group C2), 10E8v4-100fA (space group P6_422_), 10E8v4-P100gA and 10E8v4-P100fgAA, respectively (Figure 5A). Extensive hydrogen bonding networks were observed between the CDR H3 and the CDR L1 of 10E8v4, involving heavy chain residues, Tyr98, Ser100c, Pro100g, Gly100h and Glu100i, and light chain residues Ser30, Tyr32, Lys51 and Arg91. However, these hydrogen bonds were not retained in the Pro to Ala variants, suggesting the YPP motif to be critical for coordinating these hydrogen bonds, and disruption of these hydrogen-bonding interactions correlated with the degree that the CDR H3 conformations differed from that of wild type, their reduced binding affinities, and their reduced neutralization potencies (Figure 5B–D).

To assess the contribution of the hydrogen bond to the SEC behavior of 10E8v4, we substituted Tyr32_LC_ with Ala or various amino acids to disrupt the hydrogen bond between Tyr32_LC_ and Ser100c _HC_ at the tip of CDR H3, and observed that the disruption of the hydrogen bond resulted in a single SEC profile on Superdex 200 increase 10/300 GL column (GE Healthcare) and reduced potency as well (Figure S8B,C). In addition, as Masiero et al. [18] recently reported that His148_LC_ (the equivalent of His31 of 10E8v4) in a trispecific antibody [39] is an essential residue for activity, we determined the crystal structure of 10E8v4 with His31_LC_Phe mutation, and found that the hydrogen-bonding network observed in wild type was no longer maintained in the 10E8v4-H31F_LC_. As a result, its CDR H3 showed an “open” conformation deviated from that of 10E8v4 with an RMSD of 3.1 Å (Figure 5A), SEC behavior close to the wild-type, though with reduced affinity and potency (Figure 5A–C).

We also observed that the Pro to Ala mutations in the 10E8v4-100cW, a close relative to 10E8VLS (10E8v4-5R+100cF) but more potent and strongly interacting with the column matrix than 10E8VLS (Figure S9A), affected similarly the potency of 10E8v4-100cW as observed in 10E8v4 (Figure S9B), suggesting that the Pro to Ala mutations are most likely to affect the potency of 10E8VLS similarly, as the conformations of the YPP motif of 10E8v4 and 10E8VLS are nearly identical with an RMSD of 0.3 Å (Figure S9C).

Together these structure–function and correlative results indicate the conformationally dynamic cis-trans isomerization of the YPP motif to be critical for maintaining hydrogen bonding network with neighboring residues to optimally recognize its target epitope.

### 3.7. Introduction of Tyrosine-Proline to CDR H3 of 4E10 and VRC42 Results in Their Recapitulating the Multi-Peak SEC Behavior of 10E8

**Figure 6:**
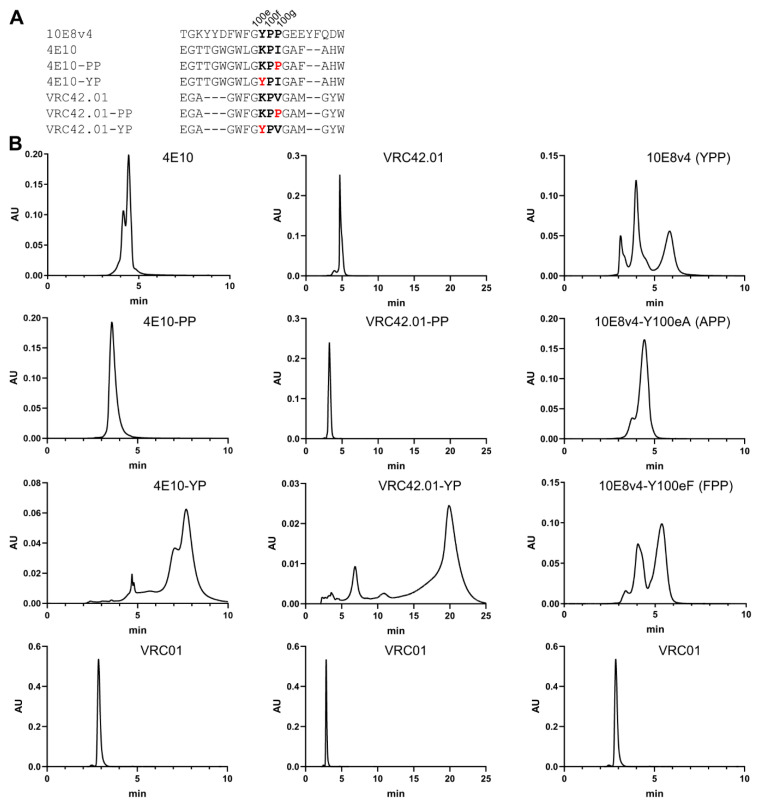
Tyr residues proceeding the Pro in the CDR H3s of antibodies 4E10 and VRC42.01 replicated the SEC anomaly observed with 10E8v4. (**A**) Aligned sequences of CDR H3s of 10E8v4, 4E10 and VRC42.01 variants. Substituted amino acids were highlighted in red. (**B**) SEC profiles of 4E10, VRC42.01 and 10E8v4 variants along with the SEC profile of VRC01 control.

To investigate the generality of cis-proline isomerization with other MPER-directed antibodies, we introduced Tyr-Pro or Pro-Pro motif to the CDR H3s of 4E10 and VRC42, and performed SEC. Both of these antibodies have a single proline in their CDR H3s, and, although this proline assumes a trans-conformation [21,40], both of these antibodies showed slightly extended interactions on SEC and evidence of multi-peak behavior (Figure 6). Notably, the addition of a second proline ablated both extended the SEC interaction and multi-peak behavior, while the addition of a tyrosine just before the proline led to highly extended SEC interactions and multi-peak behavior reminiscent of the SEC behavior of 10E8 (Figure 6B). Furthermore, Tyr100e to Ala mutation in 10E8v4 ablated its three-peak SEC profile, while Tyr100e to Phe mutation recapitulated the three-peak SEC prolife of 10E8 (Figure 6B). These results indicate that the amino acids with aromatic side chains before the proline cause their anomalous SEC behavior.

## 4. Discussion

10E8 is a broadly neutralizing antibody with substantial hydrophobicity within its antigen-binding region. We demonstrated here its multi-peak monomeric population behavior to be due to slow conformational isomerization of the YPP domain between two or more states that interacted with column matrices with increased hydrophobicity.

Two papers [17,18] have recently been published that provide insight into the basis of anomalous SEC behavior of 10E8. Both papers report that the YPP motif is responsible for the SEC anomaly, but with conflicting views as to which residues play the more important role in contributing to the anomalous SEC behavior. Masiero et al. propose Pro113 of 10E8v4 in a trispecific antibody (the equivalent of P100g in 10E8v4) as being the most important residue to control the isomeric states of the YPP motif of 10E8 [18]. Guttman et al., however, observe from NMR characterization coupled to pepsin-LC/MS assessments that Y_cis_P_trans_P and Y_trans_P_trans_P are predominant conformers in solution, and therefore conclude that P100f is the key residue that governs 10E8-anomalous SEC behavior [17]. Part of the data presented here favors the Masiero et al. proposal, as we found the P100gA mutation to reduce affinity to gp41 and neutralization potency substantially and the P100fA variant to retain reasonable neutralization potency against viruses tested (Figure 4 and Figure 5). Part of the data presented here, however, support the Guttman et al. conclusion that residue P100f is responsible for the anomalous SEC behavior of 10E8; specifically, when we introduced a Pro-Pro (PP) motif to antibodies 4E10 and VRC42, neither showed the anomalous SEC behavior, but the introduction of a Tyr-Pro (YP) motif recapitulated both multi-peak and delayed SEC behavior observed in 10E8 (Figure 6). Relevant to this, we note that amino acids with aromatic side chains proceeding proline can impact substantially the propensity of the proline to adopt a cis-conformation [41,42,43,44,45,46,47].

Masiero et al. also propose the light chain His148 of 10E8v4 Fab in a trispecific antibody (the equivalent of His31_LC_ in 10E8v4) to be the switch site for pH-dependent cis-trans isomerization. We observed mutation of His31_LC_ to Ala or Phe to reduce affinity to the gp41 epitope, to abrogate neutralization potency against all tested viruses, and to yield single peak SEC behavior with the CDR H3-YPP motif intact (Figure S10), in support of this proposal. However, our data do not support the hypothesis that the protonated state of His148 upon pH change from basic to acidic exerts charge repulsion to light chain Arg 208 of 10E8v4 Fab in a trispecific antibody (the equivalent of Arg91_LC_ in 10E8v4), which in turn disrupts the hydrogen bond between His 148_LC_ and Gly 114_HC_ (the equivalent of 100h_HC_ in 10E8v4) and results in the “open” CDR H3 conformation of 10E8. We observed 10E8v4 to retain high binding affinity to a gp41 scaffold at pH 5.8 (Figure S10B), suggesting its CDR H3 to be in the “closed” conformation, as “open” conformations observed in crystal structures of Pro to Ala substituted variants were associated with decreased affinity and potency (Figure 4 and Figure 5). Furthermore, mutation of Arg91_LC_ to Ala maintained a similar 3-peak SEC behavior (Figure S10) as shown in 10E8, indicating that the conformational states of Arg91_LC_ could not be attributed solely to the observed SEC behavior of 10E8. Overall, our results suggest the protonated state of His31_LC_ in acidic pH can lead to a “closed” CDR H3 conformation by strengthening the interaction between His31_LC_ and the YPP motif with the hydrogen bonds involving two carbonyl oxygens (Y100e and G100h) and a hydroxyl group from Glu100i_HC_ (Figure S10A). In line with this, the crystal structure of the ligand-free 10E8 Fab at pH 6.5 (PDB ID:5JNY) show a “closed” CDR H3 conformation with P100g in cis and an overall conformation of CDR H3 almost identical to that of 10E8v4 Fab structure in complex with gp41 peptide (Figure 5A). Furthermore, 10E8v4-5R+100cF (10E8VLS) completely abolished interaction with the column matrix at pH 10.55 (Figures S6 and S7) as was also observed with P100fA, P100gA and P100fgAA variants; these variants showed reduced affinity to gp41, reduced neutralization potency, and reduced interaction with the column matrix at pH 7.4 along with an “open” CDR H3 conformation (Figure 4 and Figure 5), suggesting the “open” state to be associated with basic pH.

Crystal structures of P100fA, P100gA, P100fgAA and H31_LC_F variants all showed “open” CDR H3 conformations, which correlated with reduced affinity to gp41, reduced neutralizing potency against select viruses, and reduced affinity to the column matrix (Figure 4 and Figure 5). Guttman et al. also show the binding competent population of 10E8 to the gp41 epitope to more strongly interact with the column matrix. Thus, the cis-trans conformational isomerization of the YPP motif appears to provide a mechanistic basis for CDR H3 conformational heterogeneity, with degrees of hydrophobicity confirmed by HIC, and we explain how arginine at 100 mM and pH of 10.55 could act as comodifiers to minimize successfully hydrophobic differences within the 10E8 CDR H3s. Furthermore, the crystal structures of di-proline variants, 100fA, 100gA and 100fgAA, and light chain variant, His31Phe, revealed how the YPP motif and the hydrogen bonds between the CDR H3 and the CDR L1 contributed to make the alternative conformation of the CDR H3 incompatible with gp41 recognition, providing the structural basis for the reduced binding affinity to the gp41 MPER peptide and the reduced potency against select viruses.

Overall, this study provides key mechanistic insights for 10E8 SEC method development, which was a challenging analyte with its unique multiple states of hydrophobicity. This work may be helpful in other cases when a molecule displays secondary interactions that interfere with the primary SEC separation goal of monitoring aggregation.

The reduced neutralization that we observed from altering CDR H3 flexibility, either by fixing the CDR H3 in its antigen-bound conformation with a disulfide bond or by altering prolines in a key di-proline motif, suggest the di-proline-containing CDR H3 to be functionally optimized. We propose the cis conformation of the proline to provide the hydrophobicity needed to bind tightly to its target MPER epitope and the trans conformation of the proline to be less hydrophobic and to allow more efficient scanning of antigenic surfaces. Overall, our results show how a conformationally dynamic cis-proline and the interaction between the CDR H3 and the CDR L1 of 10E8 appear to provide exactly the requisite degree of structural plasticity needed for a highly hydrophobic surface to recognize an MPER epitope.

## Figures and Tables

**Table 1:** Data collection and refinement statistics.

	10E8v4-P100fA	10E8v4-P100fA	10E8v4-P100gA	10E8v4-P100fA-P100gA	10E8v4-H31F_LC_
**PDB accession code**	7MF9	7MF8	7MF7	7MFA	7MFB
**Data collection**					
Space group	C2	P6_422_	C2	P2	C2
Cell constants					
*a, b, c (Å)*	160.9, 141.3, 137.4	42.0, 149.7, 160.2	220.2, 43.6, 226.5	95.8, 53.8, 328.5	141.8, 56.2, 69.2
α, b, g (°)	90.0, 1, 109.2, 90.0	90.0, 90.0, 90.0	90.0, 115.3, 90.0	90.0, 90.6, 90.0	90, 100.8, 90
Unique reflections	24,124	36,152	127,973	78,164	48,111
Wavelength (Å)	1	1	1	1	1
Resolution (Å)	50.0–3.70 (3.76–3.70) *	50.0–2.20 (2.24–2.20)	50.0–2.0 (2.04–2.0)	50.0–2.77 (2.85–2.77)	50–1.75 (1.78–1.75)
*R_merge_*	18.1 (54.1)	8.5 (7.2)	9.5 (86.4)	8.7(67.5)	10.0 (59.6)
R_pim_	13.6 (44.6)	3.0 (33.0)	4.7 (49.4)	6.4 (49.8)	6.1 (31.6)
CC_1/2_	0.959 (0.646)	0.999 (0.887)	0.992 (0.504)	0.990 (0.681)	0.955 (0.826)
*I/sI*	3.98 (1.05)	36 (2.04)	25.5 (1.38)	12.7 (1.13)	10.3 (1.7)
Completeness (%)	77.2 (63.2)	98.9 (99.8)	97.3 (89.4)	83.4 (85.2)	88.2 (87.3)
Redundancy	2.3 (1.7)	8.9 (8.4)	5.1 (3.7)	2.3 (2.2)	3.3 (3.4)
**Refinement**					
Resolution (Å)	40.2–3.70	32.2–2.20	36.4–2.0	35.0–2.77	40–1.75
Reflections used in refinement	24,107	36,120	127,952	78,160	39,342
*R_work_/R_free_ (%)*	24.1/26.9	20.7/23.7	20.3/23.1	23.8/28.6	21.5/24.5
No. atoms					
Protein	16,398	3259	13,163	19,637	3320
Water	0	96	674	82	442
*B-factors (Å^2^)*					
Protein	95.6	65.1	51.9	99.4	24.3
Water		65.3	52.7	66.2	38.6
R.m.s. deviations					
Bond lengths (Å)	0.02	0.02	0.006	0.004	0.012
Bond angles (°)	0.493	0.578	0.906	0.681	1.315
Ramachandran					
Favored regions (%)	97.2	98.6	97.9	97.9	98.2
Allowed regions (%)	2.8	1.4	1.9	2.2	1.8
Disallowed regions (%)	0	0	0.2	0	0

* Values in parentheses are for highest-resolution shell.

## Supplementary Materials

The following are available online at https://www.mdpi.com/article/10.3390/antib10020023/s1, Figure S1: Multi-Peak Population Profiles for 10E8VLS IgG and Fab. (A) Comparison of VRC01 and 10E8VLS on SEC where all peaks are confirmed to be of monomer mass by SEC-MALS. (B) 10E8VLS Fab showed predominantly two peaks by SEC method, with Peak 2 the dominant peak. (percentage of less dominant peaks prior to Peak 1 (5%) are not listed in the summary table). (C) Summary of 10E8VLS Fab percent distributions shown in Figure 3B,D. (D) SEC profile of time dependent re-assortment of fractionated 10E8VLS Fab Peak 2 after 24 h at both 4 °C and room temperature. Figure S2: Schematic of 10E8 IgG and Fab Conformations and Their SEC Profiles. (A) Interconverting Fab conformations are shown, for a soluble conformation (black), with typical SEC profile and for a variant hydrophobic conformation (red); in both cases an additional Phe in the CDR H3 further enhanced hydrophobic interactions. (B) Three-peak population profiles of 10E8v4 and 10E8VLS are shown deconvoluted into molecular components. Figure S3: Chromatographic Elution Profile of Antibody 10E8VLS by SEC and Optimized HIC. (A) VRC01 profile by HIC in the dotted line: as a control, VRC01 eluted at the void time. (B) 10E8VLS profile by HIC: three major peaks each eluted at different retention times. (C) The 10E8VLS (black line) and VRC01 (dotted line) profile by traditional SEC used as a reference in comparing with the HIC profile. Figure S4: SEC and HIC Profiles of Antibody 10E8VLS Correlate. (A) SEC profiles of neat injection of 10E8VLS. (B) HIC profiles of neat injection of 10E8VLS. A1, A2 and A3: reinjection of SEC fractionated peaks 1, 2 and 3 back to SEC, three fractions were confirmed. B1, B2 and B3: reinjection of SEC fractionated peaks 1, 2, and 3 back to HIC. Figure S5: Coeffects of high pH and arginine as modifiers on 10E8VLS SEC profile. (A1–A5) Hydrophobicity profiles of single factor pH only from pH 7 to 10.55. (B1–B5) The combined factors of 100 mM arginine with different pH. Figure S6: Effect of different pHs on 10E8VLS HIC profiles in presence of 100 mM Arginine. (A) pH at 10: three-peak profiles. (B) pH at 10.25: three-peak merging started. (C) pH at 10.45: peaks further merged. (D) pH at 10.55, completely merging all monomeric peaks. Figure S7: Evaluations of different amino acids as modifiers in the SEC mobile phase. From top to bottom, the mobile phases are: (A) 2× PBS, (B) 100 mM proline in 2× PBS pH 10.55, (C) 100 mM histidine in 2× PBS pH 10.55, (D) 100 mM lysine in 2× PBS pH 10.55 and (E) 100 mM arginine in 2× PBS pH 10.55. Figure S8: The electron density maps of CDR H3s, SEC profiles, neutralization potency and breadth of 10E8v4_LC_32 variants. (A) Electron density maps. (Top row) *Fo-Fc* electron density maps of CDR H3 of 10E8v4 variants contoured at 3σ. (Bottom row) *2Fo-Fc* electron density maps of CDR H3 of 10E8v4 variants contoured at 1σ. (B) Elution profiles of Superdex 200 increase 10/300 GL (GE Healthcare) size-exclusion chromatography column. (C) Neutralization potency and breadth. Figure S9: Proline to Ala mutations in the YPP motif similarly affected the potency of 10E8v4-100cW variants as the mutations affected the potency of 10E8v4. (A) The SEC profile of 10E8v4-100cW. (B) Neutralization potency and breadth of 10E8v4-100cW variants with Pro to Ala mutations. (C) Comparison of the YPP domain of 10E8v4 and 10E8VLS. Figure S10: Interaction between the YPP motif of 10E8v4 and its light chain, its binding kinetics to a gp41 scaffold and the SEC profiles of light chain variants. (A) Close-up view of the YPP motif of 10E8 in complex with gp41 peptide. (B) 10E8v4 IgG binding to gp41 scaffold, T117-F at pH 5.8 by BLI. (C) 10E8v4 IgG binding to gp41 scaffold, T117-F at pH 10.55 in the presence of 100 mM Arg by BLI. (D) SEC profiles of 10E8v4 light chain variants. Table S1: Comparison of 10E8 and its variants.
