# Supplementary material for: Structures of HIV-1 Neutralizing Antibody 10E8 Delineate the Mechanistic Basis of Its Multi-Peak Behavior on Size-Exclusion Chromatography
Source: Antibodies (Basel). 2021 Jun 10;10(2):23. doi: 10.3390/antib10020023 (PMC8293163; doi:10.3390/antib10020023)
Supplement: Supplementary file 1 [file antibodies-10-00023-s001.zip › antibodies-1134731-supplementary.pdf]

Table S1. Comparison of 10E8 and its variants.

	10E8	10E8v4	10E8VLS (10E8v4-5R+100cF	10E8v4-100cW
Heavy chain	wt	Q3R, A61E, P62E, E64K, L72D, S74T, I75K, F77T, L82cV, M84T, S87T, L89Y, R105Q, T110I	Q3R, V5R, A61E, P62E, E64K, L72D, S74T, I75K, F77T, L82cV, M84T, S87T, L89Y, S100cF, R105Q, T110I	Q3R, A61E, P62E, E64K, L72D, S74T, I75K, F77T, L82cV, M84T, S87T, L89Y, S100cW, R105Q, T110I
Light chain	wt	S1A, Y2S, E7D, T8P, G9A, G15K, R16Q, I45V, V58I, S76T, D83E, E85D	S1A, Y2S, E7D, T8P, G9A, G15K, R16Q, I45V, V58I, S76T, D83E, E85D	S1A, Y2S, E7D, T8P, G9A, G15K, R16Q, I45V, V58I, S76T, D83E, E85D
Geometric mean IC50 (IC80)(µg/ml)	0.315 (1.52)[1]	0.329 (1.67)[2]	0.044 (0.175)[3]	0.011 (0.116)[3]
Property	Sub-optimal solubility	Solubility enhanced with the potency of 10E8 maintained	Potency and solubility enhanced, observed tissue reactogenicity in human trials	Potency enhanced, not optimal physical characteristics
Reference	Huang et al. (2012) [1]	Kwon et al. (2016) [3]	Kwon et al. (2018) [2]	Kwon et al. (2018) [2]

Total number of viruses in panel: 200 study [1], 203 study [2], 208 study [3].

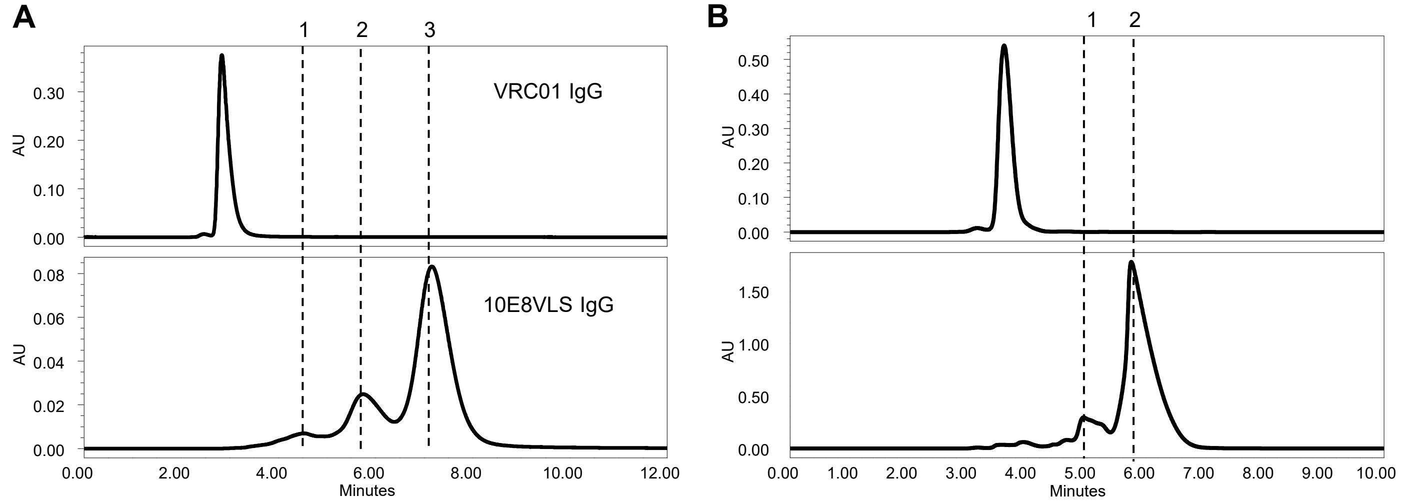

C

%Area	Peak 1	Peak 2
10E8VLS Fab	12	83
T=0 hr	4	96
T=24hr RT	13	87
T=24hr 4C	15	85

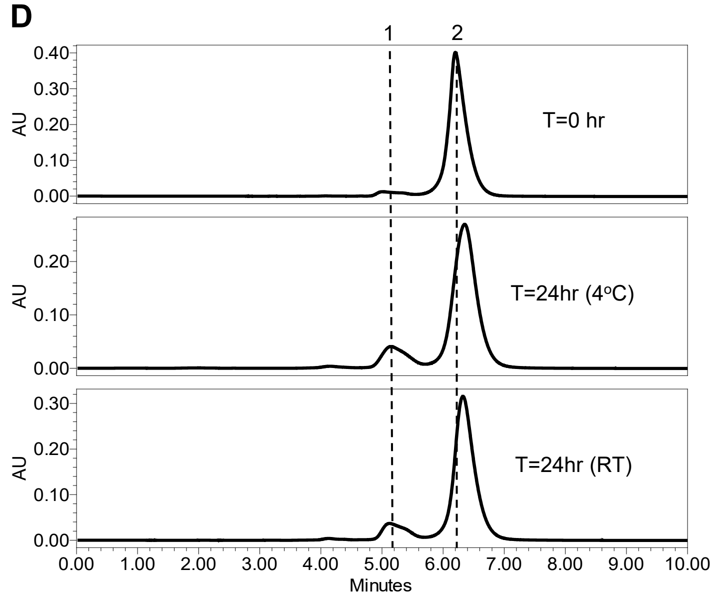

Figure S1. Multi-Peak Population Profiles for 10E8VLS IgG and Fab.

(A) Comparison of VRC01 and 10E8VLS on platform SEC where all peaks are confirmed to be of monomer mass by SEC-MALS.

(B) 10E8VLS Fab showed predominantly two peaks by SEC method, with Peak 2 the dominant peak. (percentage of less dominant peaks prior to Peak 1 (~5%) are not listed in the summary table).

(C) Summary of 10E8VLS Fab percent distributions shown in Figures 3B and 3D.

(D) SEC profile of time dependent re-assortment of fractionated 10E8VLS Fab Peak 2 after 24 hours at both 4°C and room temperature.

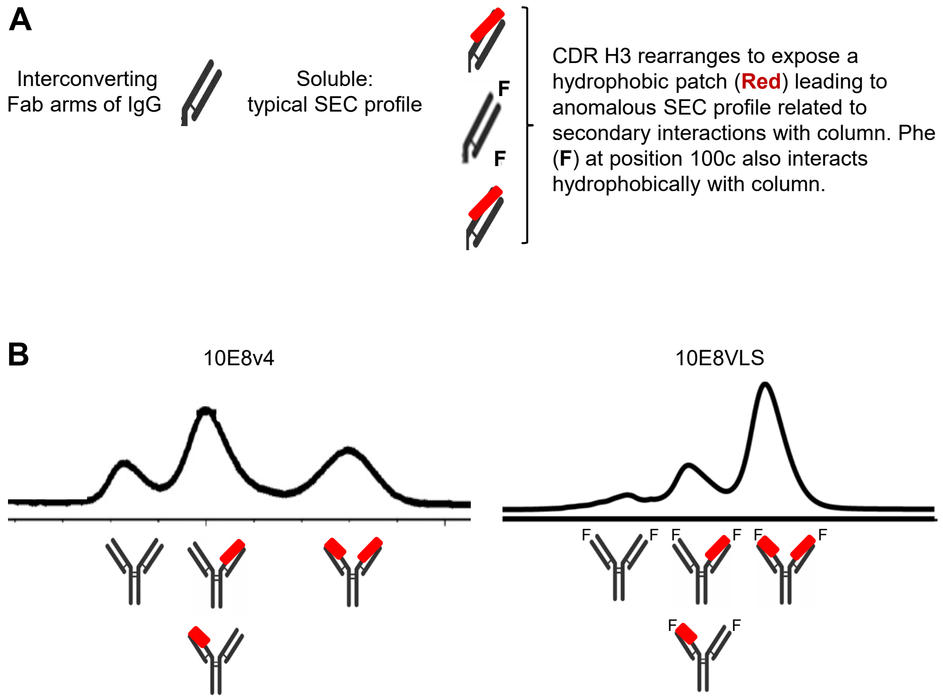

Figure S2. Schematic of 10E8 IgG and Fab Conformations and Their SEC Profiles.

(A) Interconverting Fab conformations are shown, for a soluble conformation (black), with typical SEC profile and for a variant hydrophobic conformation (red); in both cases an additional Phe in the CDR H3 further enhanced hydrophobic interactions.

(B) Three-peak population profiles of 10E8v4 and 10E8VLS are shown de-convoluted into molecular components.

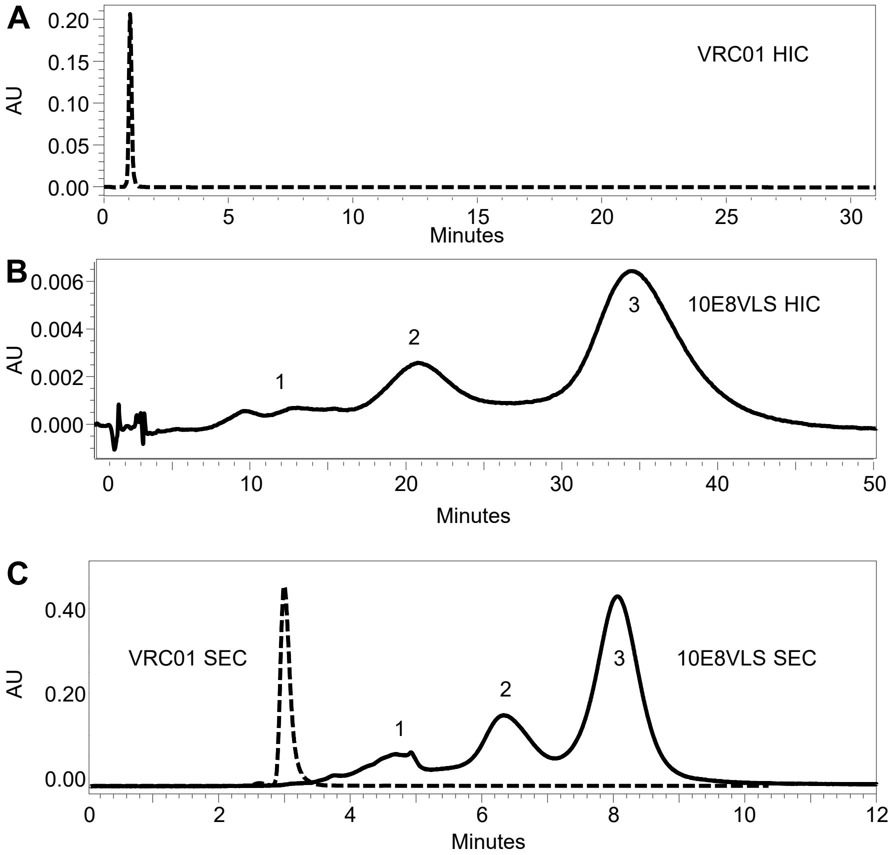

Figure S3. Chromatographic Elution Profile of Antibody 10E8VLS by SEC and Optimized HIC.
(A) VRC01 profile by HIC in dotted line: as a control, VRC01 eluted at the void time.
(B) 10E8VLS profile by HIC: three major peaks each eluted at different retention time.
(C) 10E8VLS (black line) and VRC01 (dotted line) profile by traditional SEC used as a reference in comparing with HIC profile.

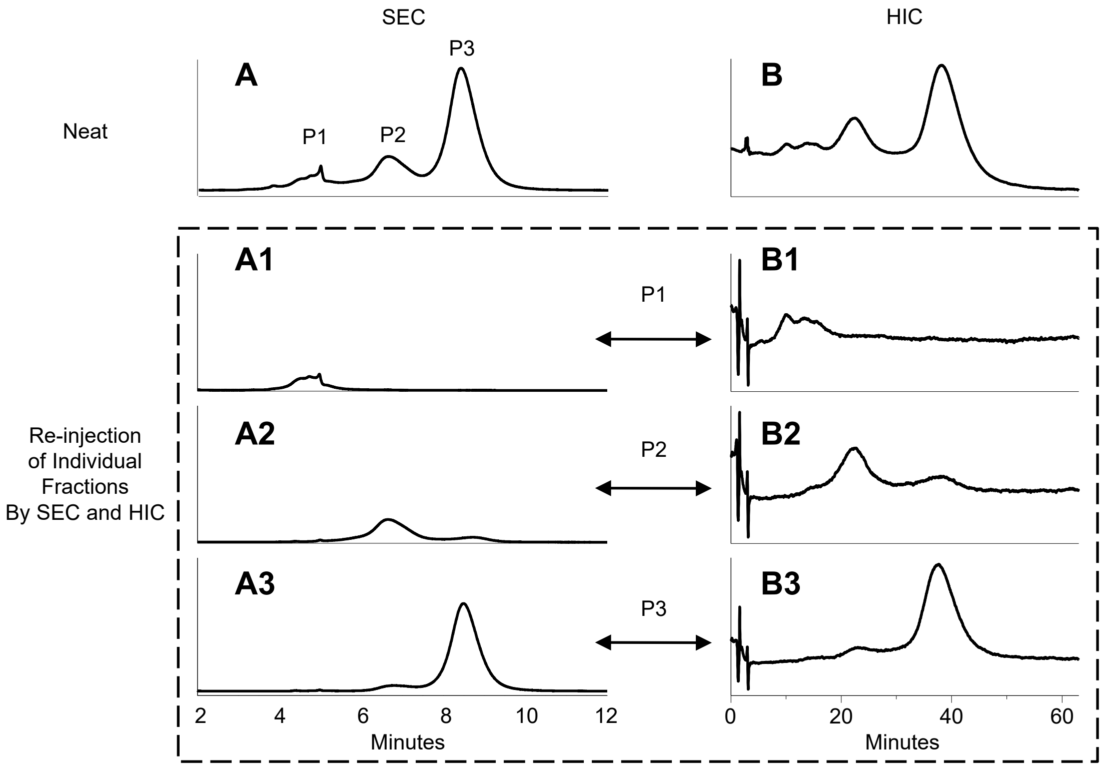

Figure S4. SEC and HIC Profiles of Antibody 10E8VLS Correlate.

(A) SEC profiles of neat injection of 10E8VLS.

(B) HIC profiles of neat injection of 10E8VLS. A1, A2 and A3: re-injection of SEC fractionated peaks 1, 2 and 3 back to platform SEC, three fractions were confirmed. B1, B2 and B3: re-injection of SEC fractionated peaks 1, 2, and 3 back to HIC.

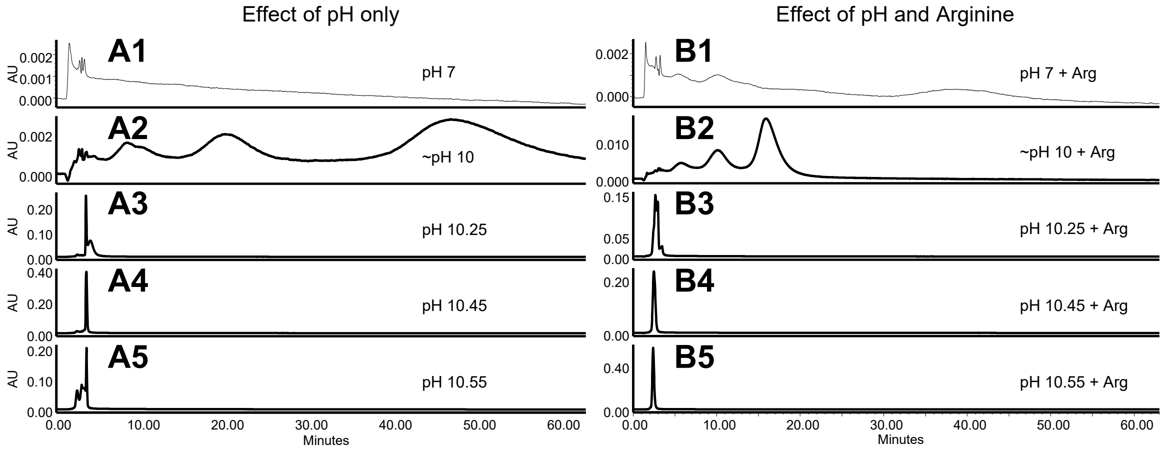

Figure S5. Co-effects of high pH and arginine as modifiers on 10E8VLS HIC profile.
(A1-A5) Hydrophobicity profiles of single factor pH only from pH 7 to pH 10.55
(B1-B5) The combined factors of 100mM arginine with different pH.

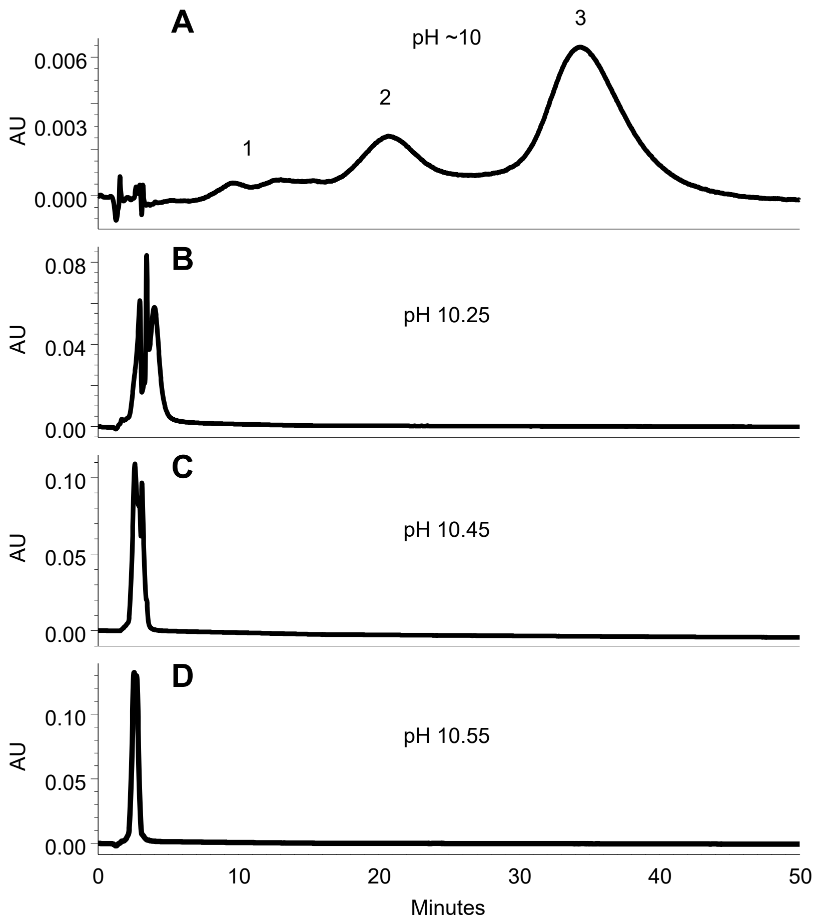

Figure S6. Effect of different pHs on 10E8VLS HIC profiles in presence of 100 mM Arginine.
(A) pH at ~10: three-peak profiles.
(B) pH at 10.25: three-peak merging started.
(C) pH at 10.45: peaks further merged.
(D) pH at 10.55, completely merging all monomeric peaks.

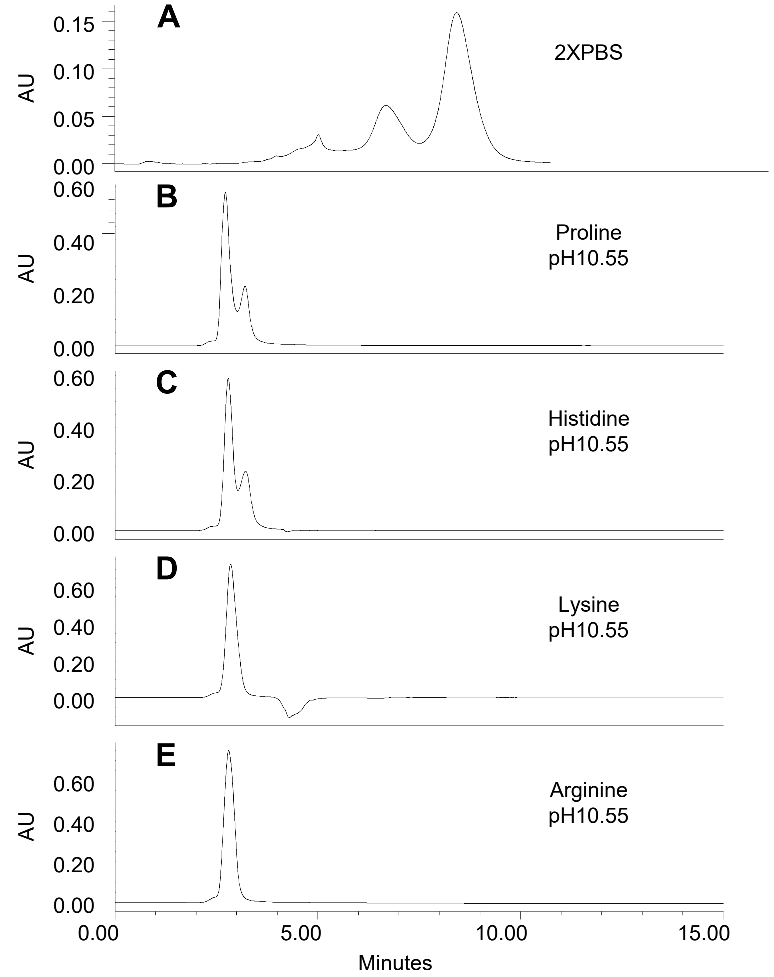

Figure S7. Evaluations of different amino acids as modifiers in SEC mobile phase.
From top to bottom, the mobile phases are:
(A) 2XPBS.
(B) 100 mM proline in 2X PBS pH10.55.
(C) 100 mM histidine in 2X PBS pH10.55.
(D) 100 mM lysine in 2X PBS pH10.55.
(E) 100 mM arginine in 2X PBS pH10.55.

A

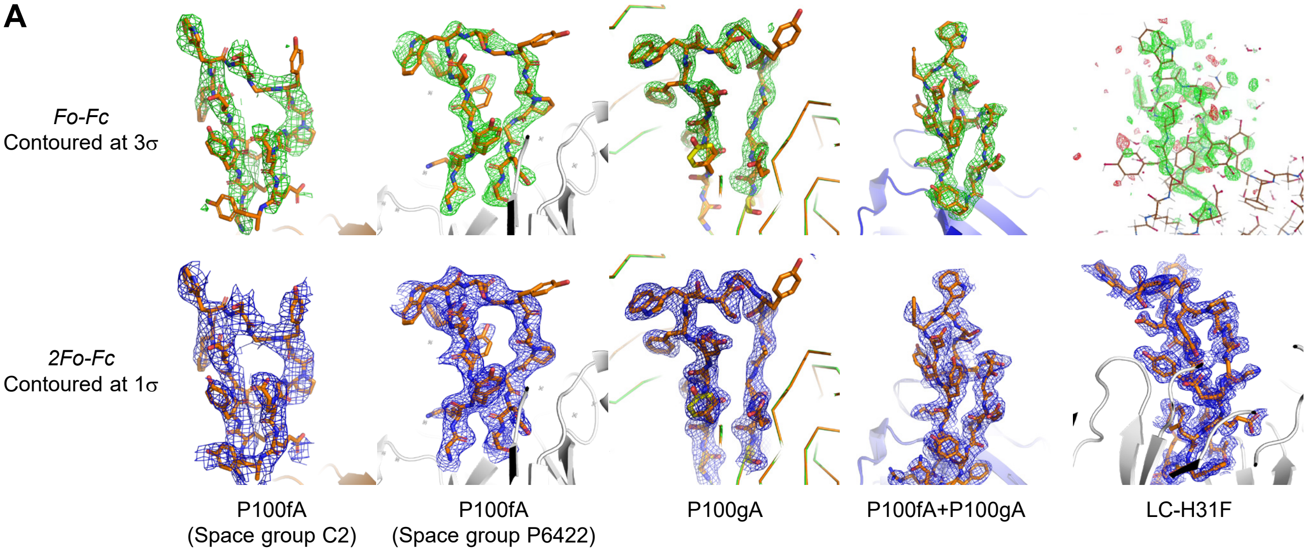

B

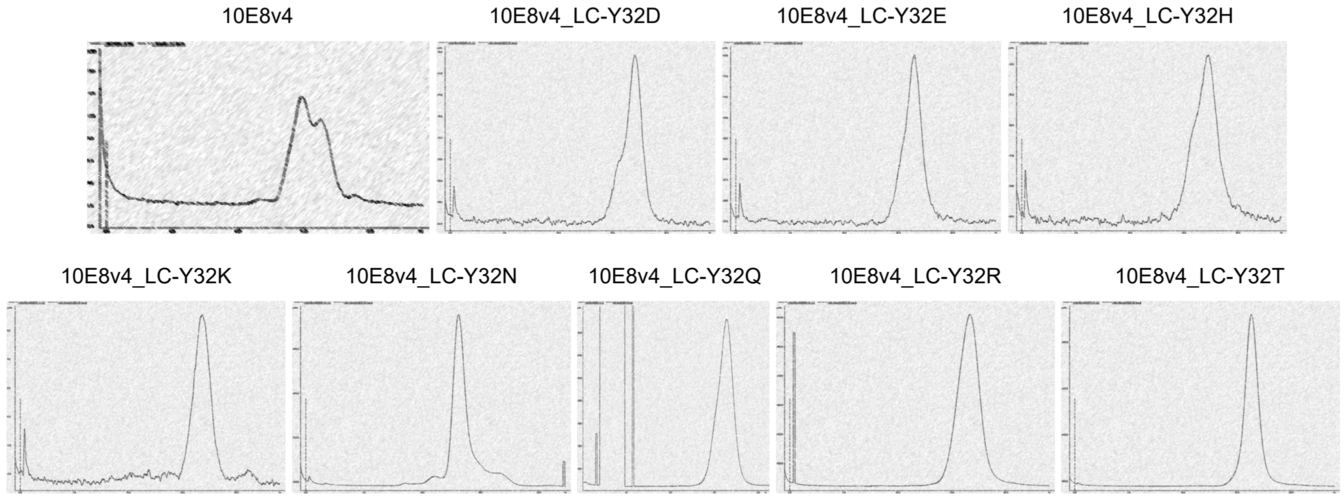

C

Clade	Virus	10E8v4	10E8v4_ LC_32D	10E8v4_ LC_32E	10E8v4_ LC_32H	10E8v4_ LC_32K	10E8v4_ LC_32N	10E8v4_ LC_32Q	10E8v4_ LC_32R	10E8v4_ LC_32T
A	KER2008.12.SG3	>50	>50	>50	>50	>50	>50	>50	>50	>50
ACD	6095.V1.C10.SG3	0.001	1.550	0.215	0.032	0.359	2.920	0.281	0.152	0.145
AE	TH966.8.SG3	0.021	>50	19.000	0.788	10.200	>50	11.500	4.080	3.760
B	6101.10.SG3	0.007	>50	>50	7.400	>50	>50	>50	>50	>50
B	PVO.04.SG3	1.130	>50	>50	>50	>50	>50	>50	>50	>50
B	YU2.DG.SG3	0.386	>50	>50	8.760	>50	>50	>50	>50	43.500
C	CNE31.SG3	1.160	>50	>50	29.900	>50	>50	>50	>50	>50
C	ZM106.9.SG3	>50	>50	>50	>50	>50	>50	>50	>50	>50
C	ZM215.8.SG3	0.013	>50	>50	1.390	33.000	>50	>50	7.520	9.250
Median IC50 (μg/ml)		0.021	1.550	9.608	4.395	10.200	2.920	5.891	4.080	6.505
Geometric Mean		0.048	1.550	2.021	2.020	4.944	2.920	1.798	1.671	3.849

Figure S8. The electron density maps of CDR H3s, SEC profiles, neutralization potency and breadth of 10E8v4_LC_32 variants.

(A) Electron density maps. (Top row) *Fo-Fc* electron density maps of CDR H3 of 10E8v4 variants contoured at 3σ. (Bottom row) *2Fo-Fc* electron density maps of CDR H3 of 10E8v4 variants contoured at 1σ.

(B) Elution profiles of Superdex 200 increase 10/300 GL (GE Healthcare) size-exclusion chromatography column.

(C) Neutralization potency and breadth.

A

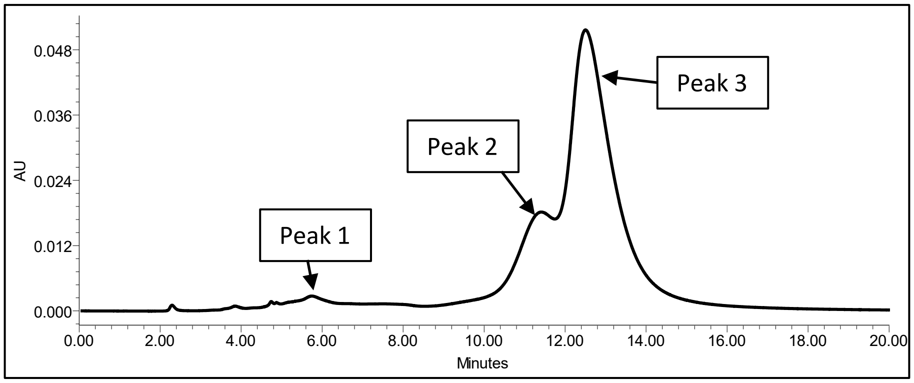

Peak	Retention Time	Area	% Area	Height
1	5.746	360196	6.35	2625
2	11.417	1209532	21.33	17993
3	12.505	4101617	72.32	51455

B

Clade	Virus	10E8v4-100cW		10E8v4-100cW-100fA		10E8v4-100cW-100gA		10E8v4-100cW-100fgAA	
		IC50	IC80	IC50	IC80	IC50	IC80	IC50	IC80
A	KER2008.12.SG3	25.9	>50	>50	>50	>50	>50	>50	>50
A	RW020.2.SG3	0.027	0.165	0.678	5.71	>50	>50	>50	>50
AE	CM244.ec1.SG3	0.005	0.081	>50	>50	>50	>50	>50	>50
B	CAAN.A2.SG3	0.197	1.00	>50	>50	>50	>50	>50	>50
B	PVO.04.SG3	0.049	0.328	0.263	1.66	16.6	>50	>50	>50
B	YU2.DG.SG3	0.038	0.349	0.133	1.16	6.64	>50	24.4	>50
C	CNE31.SG3	0.078	0.235	0.287	0.901	10.5	17.5	>50	>50
C	ZM106.9.SG3	>50	>50	>50	>50	>50	>50	>50	>50
C	ZM55.28a.SG3	0.150	0.508	0.340	1.10	11.8	31.1	>50	>50
	Median	0.064	0.328	0.287	1.160	11.150	24.300	24.400	>50
	Geometric Mean	0.105	0.292	0.297	1.612	10.810	23.329	24.400	>50

C

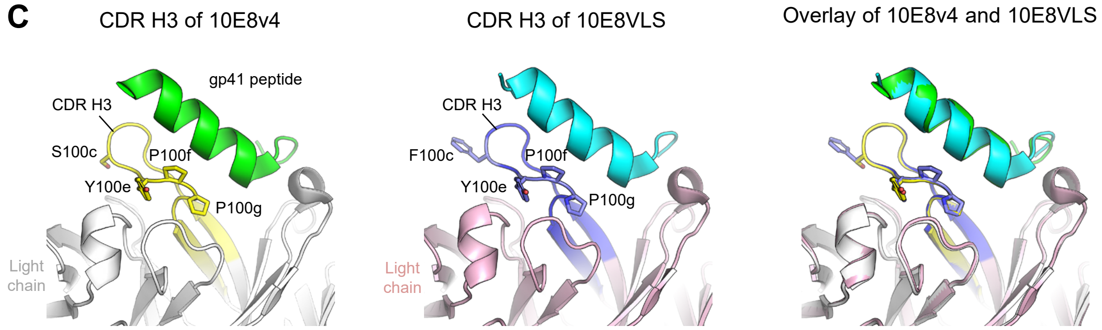

Figure S9. Proline to Ala mutations in the YPP motif similarly affected the potency of 10E8v4-100cW variants as the mutations affected the potency of 10E8v4.

(A) The SEC profile of 10E8v4-100cW.

(B) Neutralization potency and breadth of 10E8v4-100cW variants with Pro to Ala mutations.

(C) Comparison of the YPP domain of 10E8v4 and 10E8VLS.

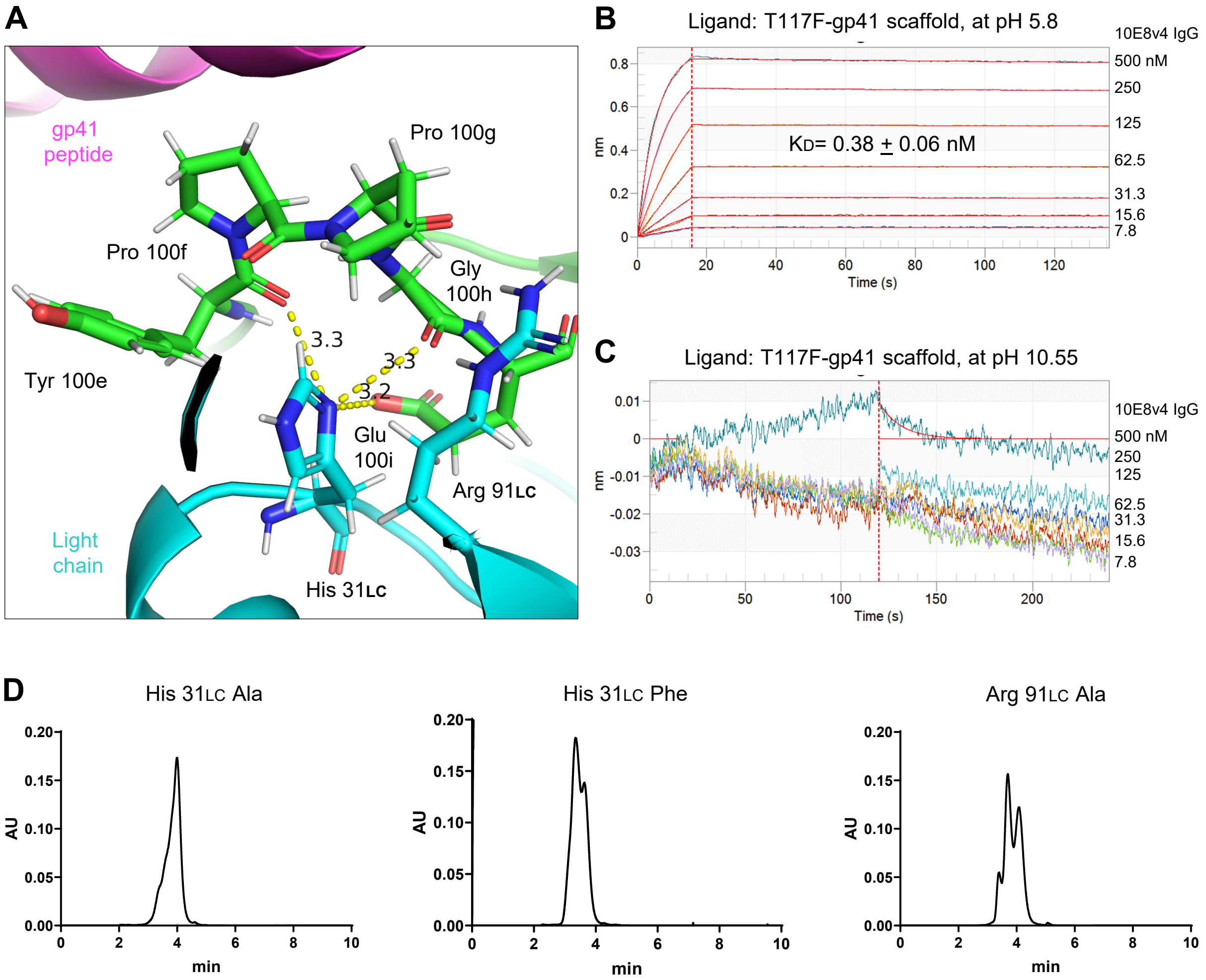

Figure S10. Interaction between the YPP motif of 10E8v4 and its light chain, its binding kinetics to a gp41 scaffold and the SEC profiles of light chain variants.

(A) Close-up view of the YPP motif of 10E8 in complex with gp41 peptide.

(B) 10E8v4 IgG binding to gp41 scaffold, T117-F at pH 5.8 by BLI.

(C) 10E8v4 IgG binding to gp41 scaffold, T117-F at pH 10.5 in the presence of 100 mM Arg by BLI.

(D) SEC profiles of 10E8v4 light chain variants.
